# Nonsense-mediated mRNA decay and metal ion homeostasis and detoxification in *Saccharomyces cerevisiae*

**DOI:** 10.1007/s10534-022-00450-0

**Published:** 2022-10-18

**Authors:** Xinyi Zhang, Bessie W. Kebaara

**Affiliations:** Department of Biology, Baylor University, One Bear Place #97388, Waco, TX 76798 USA

**Keywords:** Nonsense-mediated mRNA decay (NMD), Metal ions, Magnesium, Zinc, Copper, Iron

## Abstract

The highly conserved Nonsense-mediated mRNA decay (NMD) pathway is a translation dependent mRNA degradation pathway. Although NMD is best known for its role in degrading mRNAs with premature termination codons (PTCs) generated during transcription, splicing, or damage to the mRNAs, NMD is now also recognized as a pathway with additional important functions. Notably, NMD precisely regulates protein coding natural mRNAs, hence controlling gene expression within several physiologically significant pathways. Such pathways affected by NMD include nutritional bio-metal homeostasis and metal ion detoxification, as well as crosstalk between these pathways. Here, we focus on the relationships between NMD and various metal homeostasis and detoxification pathways. We review the described role that the NMD pathway plays in magnesium, zinc, iron, and copper homeostasis, as well as cadmium detoxification.

## The NMD pathway

The highly conserved Nonsense-mediated mRNA degradation (NMD) pathway is a translation dependent mRNA degradation pathway. Although it is best known as a surveillance pathway that degrades mRNAs with premature termination codons (PTCs) due to errors during transcription, splicing, or damage to the mRNA, NMD is now also recognized to possess additional functions. Previous studies have elucidated NMD as a regulatory pathway for gene expression through controlling the expression of fully functional protein-coding natural mRNAs. The targeting of natural mRNAs by NMD has been observed in multiple organisms including *Saccharomyces cerevisiae*, *Drosophila melanogaster*, *Arabidopsis, Caenorhabditis elegans*, and humans (Cheng and Maquat 1993; Drechsel et al. 2013; Fribourg et al. 2003; Karousis and Mühlemann 2022; Kim and Maquat 2019; Kurosaki et al. 2019; Peccarelli and Kebaara 2014; Peltz et al. 1993).

The proteins that are required for NMD to function are three highly conserved up-frameshift (UPF) factors: Upf1, Upf2 and Upf3 proteins. (Cui et al. 1995; He and Jacobson 1995; Sun et al. 1998). Involvement of these three proteins in NMD was originally identified in *S. cerevisiae* (He et al. 1993; He et al. 1997; Leeds et al. 1991) and later found in multicellular eukaryotes (Hodgkin et al. 1989; Pulak and Anderson 1993; Serin et al. 2001; Sun et al. 1998). In *C. elegans* and other multicellular organisms, NMD is regulated by Suppressors with Morphological effects on Genitalia (SMG) factors (Pulak and Anderson 1993). SMG1, SMG5, SMG6, SMG7, SMG8, and SMG9 perform a variety of functions including regulating the phosphorylation status of Upf1p (Cali et al. 1999; Grimson et al. 2004; Ohnishi et al. 2003; Yamashita et al. 2009).

In *S. cerevisiae*, hundreds of endogenous RNA Polymerase II transcripts achieve steady-state levels that are dependent on NMD. Changes in the accumulation of NMD-sensitive transcripts are sometimes associated with a change in the rate of mRNA decay. Transcripts that behave in this manner are referred to as direct targets of NMD. For others, NMD-sensitive changes in steady-state accumulation are not always accompanied by altered decay rates. These transcripts are referred to as indirect targets of NMD (Guan et al. 2006). A model accounting for indirect targets was proposed on the basis that mRNAs encoding several transcription factors are sensitive to NMD. Changes in the abundance of these transcription factors might cause changes in the rates of transcription of downstream-regulated genes in NMD-deficient cells, which could indirectly affect their abundance (Dahlseid et al. 2003).

*S. cerevisiae* has been extensively used as a model to study the regulation of natural mRNAs by NMD and several NMD targeting features have been described in previous studies. Characterized NMD-inducing features in *S. cerevisiae* include inefficiently or nonproductive alternatively spliced pre-mRNAs (He et al. 1993; Kawashima et al. 2014), mRNAs containing atypically long 3′ untranslated regions (UTRs) (Kebaara and Atkin 2009; Muhlrad and Parker 1999), mRNAs containing upstream open reading frames (uORFs) (Gaba et al. 2005; Guan et al. 2006), mRNAs subject to programmed ribosomal frameshifting, and mRNAs subject to out-of-frame translation initiation caused by leaky ribosomal scanning (Guan et al. 2006; Parker 2012; Welch and Jacobson 1999).

The abovementioned NMD targeting features have been found to target mRNAs to NMD in multiple organisms (Peccarelli and Kebaara 2014). A highly conserved NMD targeting mechanism is the presence of atypically long 3′-UTRs (Kebaara and Atkin 2009; Peccarelli and Kebaara 2014). In *S. cerevisiae*, mRNA 3′-UTRs are fairly short and typically range in size from 50 to 200 nucleotides (nts) with a median size of ~ 121 nts (Graber et al. 2002). In general, mRNAs with 3′-UTRs that are 350 nts or longer are considered atypically long and are likely to be regulated by NMD (Deliz-Aguirre et al. 2011; Kebaara and Atkin 2009). Furthermore, some mRNAs produce different isoforms of the same mRNA that vary in their 5′ and 3′-UTR lengths. These mRNA isoforms may have different sensitivity to NMD. Genes produce multiple transcripts that vary in the 3′-UTR lengths because of alternative 3′-end processing. Moreover, mRNA 3′-end processing can be sensitive to growth conditions, and varying environmental conditions may produce mRNA isoforms of varying lengths (Kim Guisbert et al. 2007). Thus, genes that produce mRNA isoforms of different 3′-UTR length may generate one form of the transcript that is degraded by NMD while the other may not be regulated by the pathway (Peccarelli et al. 2016).

In addition to 3′-end processing, transcript heterogeneity at the 5′-end is particularly relevant for NMD targeting. The use of different transcription start sites (TSSs) may generate mRNAs with distinct 5′-UTRs that could include uORFs. uORFs are potent regulatory elements located in 5′-UTRs of transcripts that can inhibit the translation of the downstream main ORF, but some uORFs enhance expression. If the uORF of an mRNA is translated, the stop codon can be recognized as a PTC, resulting in degradation of the mRNA by NMD. However, several naturally occurring uORF-containing transcripts are resistant to NMD. For example, yeast *GCN4* and *YAP1* contain uORFs and are resistant to NMD because they contain stabilizer elements (STEs) in the 5′-UTR that inactivate NMD (Ruiz-Echevarría et al. 1998; Vilela et al. 1998). Pub1p was identified as the factor that interacts with the leader region of the *YAP1* transcript (Ruiz-Echevarría and Peltz 2000). STE-containing transcripts are destabilized in the *pub1* ∆ strain (Ruiz-Echevarría and Peltz 2000). On the other hand, *CPA1* mRNA containing uORF is sensitive to NMD pathway (Gaba et al. 2005; Guan et al. 2006; F. He et al. 2003). Thus, transcripts containing uORFs may be substrates for NMD mediated degradation while the ones containing STEs may not be regulated by the pathway.

In addition to containing NMD-targeting features that activate their degradation, regulation of natural mRNAs occurs in specific cellular contexts and environmental conditions. Multiple conditions affect NMD-mediated degradation of mRNAs. Here we focus on NMD-mediated control of genes involved in bio-metal homeostasis and metal ion detoxification. We review the described role NMD plays in magnesium, zinc, iron, and copper homeostasis and cadmium detoxication.

## Magnesium, zinc, copper, and iron homeostasis in yeast

Bio-metals are essential for normal cellular functions but can be highly toxic. Organisms have evolved subtle mechanisms to utilize essential metals and to detoxify excess toxic levels. Magnesium (Mg^2+^) is an essential metal that serves as a cofactor for many cellular enzymes and is required for normal cellular growth. Yeast cells regulate cytoplasmic magnesium levels and store magnesium in the vacuole and mitochondria. In yeast, magnesium homeostasis is regulated by the controlled activity of magnesium uptake transporters in the plasma membrane and transporters responsible for intracellular magnesium storage. *ALR1* and *ALR2* were the first magnesium transporters identified on the plasma membrane (MacDiarmid and Gardner 1998). Yeast strains lacking *ALR* gene activity require additional magnesium for growth, and expression of either *ALR1* or *ALR2* corrects the magnesium requiring phenotype (MacDiarmid and Gardner 1998). An additional protein, (Mnr2p) is also required for magnesium homeostasis (Pisat et al. 2009). Mnr2p is localized to the vacuolar membrane, implicating this organelle in magnesium storage. In magnesium replete conditions, yeast cells accumulate intracellular stores of magnesium that supports growth under magnesium deplete conditions. Overexpression of *MNR2* suppresses the growth defect of an *alr1∆ alr2∆* mutant, indicating that *MNR2* could function independently of the *ALR* genes (Pisat et al. 2009). Lastly, Mme1p has been characterized as a mitochondrial magnesium exporter in yeast (Cui et al. 2015). When yeast cells are grown in magnesium deplete conditions, mitochondrial transporters reallocate mitochondrial magnesium to the cytoplasm for immediate use.

It is believed that nearly 10% of the eukaryotic proteome requires zinc to function (Bird and Wilson 2020). Zinc can function catalytically at active sites of enzymes or structurally as zinc fingers (Eide 2009). Cells have evolved homeostatic mechanisms to cope with zinc deplete or replete conditions. Transcriptional response to zinc deplete conditions is facilitated by Zap1p. Zap1p regulates target genes by binding to the Zap1 responsive elements (ZREs) in the promoters of target genes (Eide 2009). For a majority of the genes Zap1p activates transcription, but for a few genes Zap1p represses transcription (Eide 2009). A primary function of Zap1p activated genes is zinc uptake. Zinc specific transporters are encoded by *ZRT1* and *ZRT2* (Zhao and Eide 1996a, b), while *FET4* transports zinc, copper, and iron (Eide 2009). Zrt1p provides high affinity zinc uptake while Zrt2p functions in low affinity zinc uptake (Zhao and Eide 1996a, b). The expression of *ZRT1* is regulated at post-translational level as well. The post-translational inactivation of Zrt1p is important for zinc homeostasis and may be an important mechanism for preventing cadmium uptake and toxicity in zinc-limited cells (Gitan et al. 2003). Under Zinc replete conditions, yeast cells store zinc in the vacuole. This prevents excessive zinc mediated toxicity and further provides a zinc source in zinc deplete conditions. Cot1p and Zrc1p mediate zinc storage (Eide 2009). Additionally, *ZRT3* encodes a Zap1p regulated protein that transports zinc out of the vacuole in zinc deplete conditions.

Copper is essential for multiple cellular functions in eukaryotes. In yeast, it serves as an important cofactor for enzymes, including cytochrome c oxidase for respiration and Cu, Zn-superoxide dismutase (Sod1) for oxidative stress protection (Karlin 1993; Linder and Hazegh-Azam 1996). As copper is a redox-active metal with two forms, Cu^2+^ and Cu^+^, oxidative damage of macromolecules can occur when copper accumulates to excessive amounts within the cell (Halliwell and Gutteridge 1984). Copper-responsive transcriptional regulation is of importance in fungi, where a variety of factors control genes required for copper uptake and detoxification (Bird 2008). Intracellular [Cu]_free_ is limited to less than one free copper ion per cell, and a pool of free copper ions is not used in the physiological activation of metalloenzymes (Rae et al. 1999).

Iron (Fe^2+)^ is an essential element for all eukaryotes and functions as a cofactor for many enzymes involved in cellular functions including DNA replication and repair, cellular respiration, photosynthesis, oxygen transport, lipid metabolism and translation (Puig et al. 2005; Romero et al. 2021) Studies have shown that iron acquisition systems in yeast are regulated at the transcriptional and post-transcriptional levels (Martins et al. 2018). Under iron deplete conditions, yeast cells initiate iron uptake, activate intracellular iron stores, and undergo metabolic adaptations to iron deficiency. Under these conditions, yeast cells activate a group of genes known as the iron regulon which includes genes involved in high-affinity iron uptake. The yeast *AFT1* and *AFT2* paralogs are the major transcription factors that regulate the activation of genes in iron deplete conditions (Martinez-Pastor et al. 2017). Aft1p is constitutively expressed even under iron replete conditions but does not activate transcription. However, under low iron conditions, Aft1p is imported from the cytoplasm to the nucleus and activates transcription of genes required for iron uptake from the environment, mobilization of stored iron, and metabolic changes that occur under iron deplete conditions (Bird 2008). Post-transcriptional control under iron deplete conditions involves Cth1 and Cth2 proteins, two tristetraprolin family members that interact directly with target mRNAs through adenosine and uridine rich elements (AREs) in the 3′-UTRs of the mRNAs. Cth1 and Cth2 proteins downregulate mRNAs that encode proteins involved in many iron-dependent processes, thus resulting in conservation and metabolic adjustment to iron availability (Bird 2008; Puig et al. 2005, 2008).

Copper and iron homeostasis are linked and NMD plays a role in both bio-metal homeostatic mechanisms as discussed below (Deliz-Aguirre et al. 2011; Guan et al. 2006; Peccarelli et al. 2016; Peccarelli et al. 2019; Wang et al. 2013). Copper is required for iron acquisition and both metals are essential elements that function in multiple metabolic pathways. Although copper and iron are required for normal cellular function, excessive amounts of either metal can lead to oxidative damage of macromolecules. Thus, cells have evolved subtle ways for homeostatic metabolism of copper and iron. Furthermore, defects in copper and iron homeostasis are implicated in several human diseases. These include defects in growth and development, organ damage, degenerative diseases, anemia, and cancer.

### **NMD and magnesium homeostasis**

Inactivation of the NMD pathway can cause ribosome readthrough of some nonsense and frameshift mutations resulting in nonsense suppression (Wu et al. 2020). Ribosome readthrough of the three stop codons (UGA, UAA and UAG) depends on various factors, including the specific stop codon (Wu et al. 2020). To understand how NMD factors affect the efficiency of translation, Johansson and Jacobson (2010) screened for mutations that counteract nonsense suppression of NMD mutants. One complementation group identified strains with mutations in *ALR1*, which as described above, encodes a plasma membrane transporter required for efficient magnesium uptake (Graschopf et al. 2001; MacDiarmid and Gardner 1998). A mutation in the *ALR1* gene eliminates the translation termination defect of NMD deficient cells. Notably, *ALR1* and the closely related gene *ALR2* encode mRNAs that are directly regulated by NMD (Table 1) (Johansson and Jacobson 2010). The reduced translational fidelity in NMD mutants is due to increased Alr1p and Alr2p and consequently elevated intracellular level of magnesium. Magnesium influences translational fidelity because increasing magnesium concentrations increases readthrough at nonsense codons and misreading at sense codons. The reduced translational fidelity seen in NMD mutants is an indirect consequence of elevated Alr1p levels. Additionally, the NMD targeting feature of the *ALR1* mRNA was identified as a uORF (Johansson and Jacobson 2010). Thus, NMD and magnesium homeostasis are interlinked, and precise regulation of both processes is essential for normal cellular function.

**Table 1:** Genes encoding mRNAs involved in metal homeostasis and regulated by NMD

Metal ion	mRNA regulated by NMD and the function of the encoded protein	References
Magnesium	*ALR1-* Plasma membrane protein required for efficient magnesium uptake, essential gene *ALR2-* Plasma membrane protein for efficient magnesium uptake; not essential for growth	(Guan et al. 2006; Johansson et al. 2007; Johansson and Jacobson 2010)
Zinc	*ZRT1—*High-affinity zinc transporter of the plasma membrane	(Johansson et al. 2007; Toesca et al. 2011)
Copper	*CTR2-* Copper transporter of the vacuolar membrane *CTR3-* High-affinity copper transporter of the plasma membrane *MAC1-* Copper-sensing transcription factor involved in regulation of genes required for high affinity copper transport *COX19-* Protein requires for cytochrome c oxidase assembly *COX23-* Mitochondrial intermembrane space protein that functions in mitochondrial copper homeostasis, important for functional cytochrome oxidase expression *COX17-* Copper metallochaperone that delivers copper to cytochrome *c* oxidase (CcO) through two copper-binding intermembrane space-associated proteins Sco1 and Cox11 *CRS5-* Copper-binding metallothionein *PCA1-* Cadmium transporting P-type ATPase; may also have a role in copper and iron homeostasis	(Deliz-Aguirre et al. 2011; Guan et al. 2006; Johansson et al. 2007; Murtha et al. 2019; Peccarelli et al. 2016)
Cadmium	*PCA1-* Cadmium transporting P-type ATPase; may also have a role in copper and iron homeostasis	(Guan et al. 2006; Wong et al. 2021)
Iron	*FRE2*- Ferric and cupric reductase reduces siderophore-bound Fe^2+^ and oxidized copper prior to uptake by transporters. Expression induced by low Fe^2+^ levels *FRE3*- Ferric reductase; reduces siderophore-bound Fe^2+^ prior to uptake by transporters; expression induced by low Fe^2+^ levels *FRE6-* Putative ferric reductase with similarity to Fre2p; expression induced by low Fe^2+^ levels. Reduces vacuolar Fe^2+^ and copper prior to export to the cytosol *AFT2- AFT2* is a paralog of *AFT1*. *AFT2* is a transcription factor that activates genes required for Fe^2+^ homeostasis and resistance to oxidative stress *ARN1-* A ferrichrome-type siderophore transporter *ARN2-* A member of the ARN family of transporters that recognizes siderophore-iron chelates *ENB1 (ARN4)-* Ferric enterobactin transmembrane transporter *FET3-* Ferrous iron transporter *FIT1*- Cell wall mannoprotein facilitating iron uptake *FIT3*- Cell wall mannoprotein facilitating iron uptake	(Guan et al. 2006; Johansson et al. 2007; Peccarelli et al. 2019; Toesca et al. 2011)

### **NMD and zinc homeostasis**

Transcription of non-coding RNA (ncRNA) close to protein coding genes has been shown to function as a mode of transcriptional control (Neil et al. 2009). Most ncRNAs found to regulate gene expression in *S. cerevisiae* are antisense transcripts (e.g., *PHO84*, *Ty1* and *GAL10* locus) which control chromatin modification marks at these genes (Berretta et al. 2008; Camblong et al. 2007, 2009; Houseley et al. 2008). Toesca et al. (2011) showed that *S. cerevisiae* NMD mutants accumulate 5′-extended RNAs (CD-CUTs) produced from cryptic upstream transcription. Transcription of these CD-CUTs mediates repression at promoters by preventing premature binding of RNA polymerase II in conditions of metal repletion. CD-CUTs are targeted by cytoplasmic turnover pathways that include the exoribonuclease Xrn1p and the core NMD factor Upf1p. Importantly, CD-CUT transcription or accumulation also interferes with binding of the transcriptional activators *AFT1* and *ZAP1*, which modulate transcriptional accumulation of metal homeostasis mRNAs such as *FIT3* and *ZRT1* (Table 1). *FIT3* and *ZRT1* are involved in iron homeostasis and zinc homeostasis, respectively. Thus, NMD controls the accumulation of transcripts that negatively interfere with the transcription of genes involved in zinc and iron homeostasis (Toesca et al. 2011).

## The role NMD plays in copper homeostasis

**Fig. 1:**
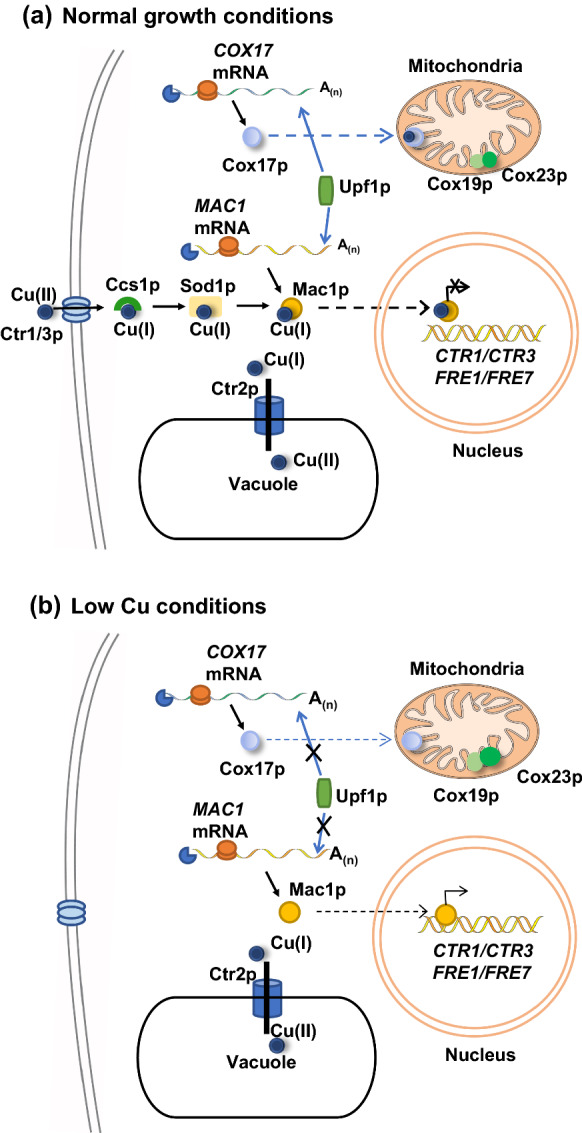
Schematic representation of condition specific NMD regulation of genes involved in copper homeostasis in *S. cerevisiae*. An arrow pointing to an mRNA from Upf1p indicates NMD regulates the mRNA, an X through the arrow indicates that NMD does not regulate the mRNA. **a** Genes involved in copper homeostasis regulated by NMD under normal growth conditions. *MAC1, COX17, COX19, COX23* are NMD targets. Copper (Cu) is delivered to Mac1p by Ccs1p and Sod1p, thus Mac1p can no longer bind to the promoter regions of *CTR1/CTR3* and *FRE1/FRE7*. The detailed mechanism of Cu delivered to Cox17p is not well elucidated. Cox17p transfers Cu to Sco1p and Cox11p and eventually delivers to cytochrome c oxidase. **b** Condition specific NMD regulation of *MAC1* and *COX17* mRNAs under low copper conditions. In low copper conditions, *MAC1* and *COX17* mRNAs escape NMD-mediated degradation*.* Therefore, more Mac1p is translated, and it is not bound to Cu^+^. Consequently, Mac1p binds to the transcription activation region of *CTR1/CTR3* and *FRE1/FRE7* genes*.* Mac1p also regulates the expression of two cell-surface ferric and cupric reductases encoded by *FRE1* and *FRE7* (L. J. Martins et al. 1998; Peña et al. 1998)

Global expression profiling studies identified genes involved in copper homeostasis as potential NMD targets in *S. cerevisiae*. These genes include *CTR2, CTR3, MAC1, COX23, CRS5, PCA1, FRE2* and *COX19* (Table 1) (Guan et al. 2006; Johansson et al. 2007). The proteins encoded by these eight mRNAs are involved in various aspects of copper homeostasis. *MAC1* is a transcription factor which plays a vital role in regulating high-affinity copper uptake system (Yamaguchi-Iwai et al. 1997). Mac1 protein comprises an N-terminal DNA-binding copper-fist domain and a C-terminal half responsible for transactivation, including two cysteine-rich sequences that bind to a total of eight Cu(I) ions (Graden and Winge 1997). Copper inactivates Mac1p due to an intramolecular interaction between the N- and C-terminal cysteine-rich motifs by the formation of a poly-copper cluster (Jensen and Winge 1998). In response to copper deplete conditions, Mac1p binds to Cu-responsive *cis*-acting (CuRE) promoter elements of target genes and activates the high-affinity copper uptake systems encoded by *CTR1* and *CTR3* (Fig. 1). (Peña et al. 1998).

The *MAC1* transcript was first identified as a potential NMD substrate because it preferentially associates with Upf1p (Johansson et al. 2007). Subsequently, it was confirmed that *MAC1* mRNA is a direct NMD target in rich media with altered decay rates in wild-type and NMD mutant strains (Peccarelli et al. 2016). *MAC1* encodes two transcripts that differ in their 3'-UTR length. Interestingly, under low copper conditions, two predominant *MAC1* transcripts are transcribed. The decay rates of both *MAC1* mRNA isoforms encoded under low copper vary as compared to the decay of *MAC1* mRNA in rich media (Peccarelli et al. 2016). Under low copper conditions, both *MAC1* mRNA isoforms evade NMD despite having atypical long 3'-UTRs (Peccarelli et al. 2016) (Fig. 1). Thus, regulation of *MAC1* mRNA isoforms by NMD is conditional, based on copper levels. Under low copper conditions NMD is functional as *CYH2* pre-mRNA which is usually used as an NMD control is regulated by NMD in low copper (Murtha et al. 2019).

An additional mRNA involved in copper homeostasis and regulated by NMD is the *CTR2* mRNA. *CTR2* encodes a copper transporter of the vacuolar membrane that controls the flux of copper into the vacuole (Rees et al. 2004). *S. cerevisiae CTR2* mRNAs have long 3′-UTRs that contribute to the degradation of the mRNAs by NMD (Peccarelli et al. 2014). Overexpression of *CTR2* generates yeast strains with an increased copper tolerance phenotype (Peccarelli et al. 2014). This phenotype was observed in NMD mutant strains with a functional Ctr2p as well (Deliz-Aguirre et al. 2011).

In *S. cerevisiae*, cytochrome c oxidase requires copper to be fully functional in mitochondrial aerobic respiration. *COX19* and *COX23* encode proteins required for cytochrome c oxidase assembly and were identified as potential NMD targets in *S. cerevisiae* (Guan et al. 2006; Johansson et al. 2007). The *COX19* transcript has an atypical long 3′-UTR that contributes to the degradation of the mRNA by NMD (Peccarelli et al. 2016). In rich media, *COX19* was found to be a direct NMD target, while *COX23* was an indirect NMD target (Peccarelli et al. 2016). *COX17* is a homologous copper metallochaperone required for the assembly of cytochrome c oxidase. Cox17p transfers copper to two proteins associated with the mitochondrial intermembrane space, Sco1p and Cox11p, which eventually deliver copper to cytochrome c oxidase (Horng et al. 2004). Cox17p, Cox19p and Cox23p are required for mitochondrial copper utilization. When yeast cells were grown in varying amounts of copper, the regulation of *COX17*, *COX19* and *COX23* mRNAs by NMD was variable (Murtha et al. 2019). Specifically, *COX19* mRNA was a direct NMD target under all conditions tested with varying copper levels. *COX17* mRNA was directly regulated by NMD only in rich media but not under low or high copper conditions (Fig. 1). *COX23* mRNA was immune to NMD under low copper conditions and was found to be an indirect target in rich media (Murtha et al. 2019).

NMD also regulates mRNAs encoding proteins involved in protection from metal toxicity. Two mRNAs found to be regulated by NMD and are involved in protection from metal toxicity are *PCA1* and *CRS5* (Peccarelli et al. 2016). Interestingly, both *CRS5* and *CUP1* genes encode metallothioneins, which bind to excessive amounts of copper in yeast cells and are differentially regulated by NMD. The major metallothionein *CUP1* is induced by the *ACE1* transcription factor when yeast cells are exposed to elevated copper levels (Evans et al. 1990). *CRS5* is an indirect NMD target under normal growth conditions, while *CUP1* is not regulated by NMD (Murtha et al. 2019; Peccarelli et al. 2016).

*PCA1* mRNA is also regulated by NMD and involved in the protection of the cell from metal toxicity (Guan et al. 2006; Wong et al. 2021) *PCA1* is an evolutionarily conserved P_1B_-type cation-transporting ATPase that is widely distributed from bacteria to humans (Rad et al. 1994). *PCA1* has been proposed to be involved in copper and iron homeostasis (De Freitas et al. 2004; Rad et al. 1994). Copper resistance mediated by *PCA1* is not dependent on catalytic activity but is understood that a cysteine-rich region located in the N-terminal sequesters excess copper. In addition to the *PCA1* allele found in most common lab yeast strains, *CAD2*, an alias of *PCA1* has been identified (Shiraishi et al. 2000). The protein encoded by *CAD2* functions in the cadmium efflux system of yeast cells (hereafter referred as *PCA1 970G*). The *PCA1* allele found in most common lab yeast strains possess a missense mutation in the ATP-binding residue conserved in P_1B_ -type ATPases [hereafter referred as *PCA1 970R*; (Adle et al. 2007; Wong et al. 2021)]. In humans, a substitution in the ATP7B similar to the one found in *PCA1* leads to Wilson's disease. Wilson's disease is characterized by excessive accumulation of copper in hepatic and neuronal tissues (Vulpe and Packman 1995).

Overall, the condition-specific regulation of mRNAs by the NMD pathway allow yeast cells to control the expression of select mRNAs in response to environmental copper conditions. The control of copper homeostasis genes occurs at multiple levels including mRNA levels via the NMD pathway. As reviewed here, the NMD pathway regulates mRNA involved with copper transport, adaptation to low copper conditions, mitochondrial copper utilization, and excess copper detoxification.

## Toxic metal detoxification

Cadmium (Cd) is a non-essential and toxic environmental contaminant (Wysocki and Tamás 2010). In *S. cerevisiae*, cadmium is detoxified by ATP-binding cassette transporters after conjugation to glutathione (Prévéral et al. 2009). Pca1p is the major cadmium detoxification mechanism in yeast. In addition to *PCA1,* yeast cadmium factor gene (*YCF1*), a vacuolar transporter, sequesters cadmium and other heavy metals as well as glutathione into the vacuole to counter cell stress (Li et al. 1996; Li et al. 1997).

In the lab strain W303a, *PCA1 970R* mRNA was identified as an indirect NMD target in rich media (Wong et al. 2021b). The decay rate of the *PCA1 970R* mRNAs was similar in wild-type and NMD mutant strains (Guan et al. 2006; Peccarelli et al. 2016). The *PCA1* gene encodes two major transcripts. The longer *PCA1* transcript has an atypical long 3'-UTR of 650 nts and may be subject to programmed ribosomal frameshifting. These are two known NMD targeting features (Belew et al. 2011; Kebaara and Atkin 2009), suggesting that *PCA1* may be differently regulated by NMD depending on different environmental conditions.

**Fig. 2:**
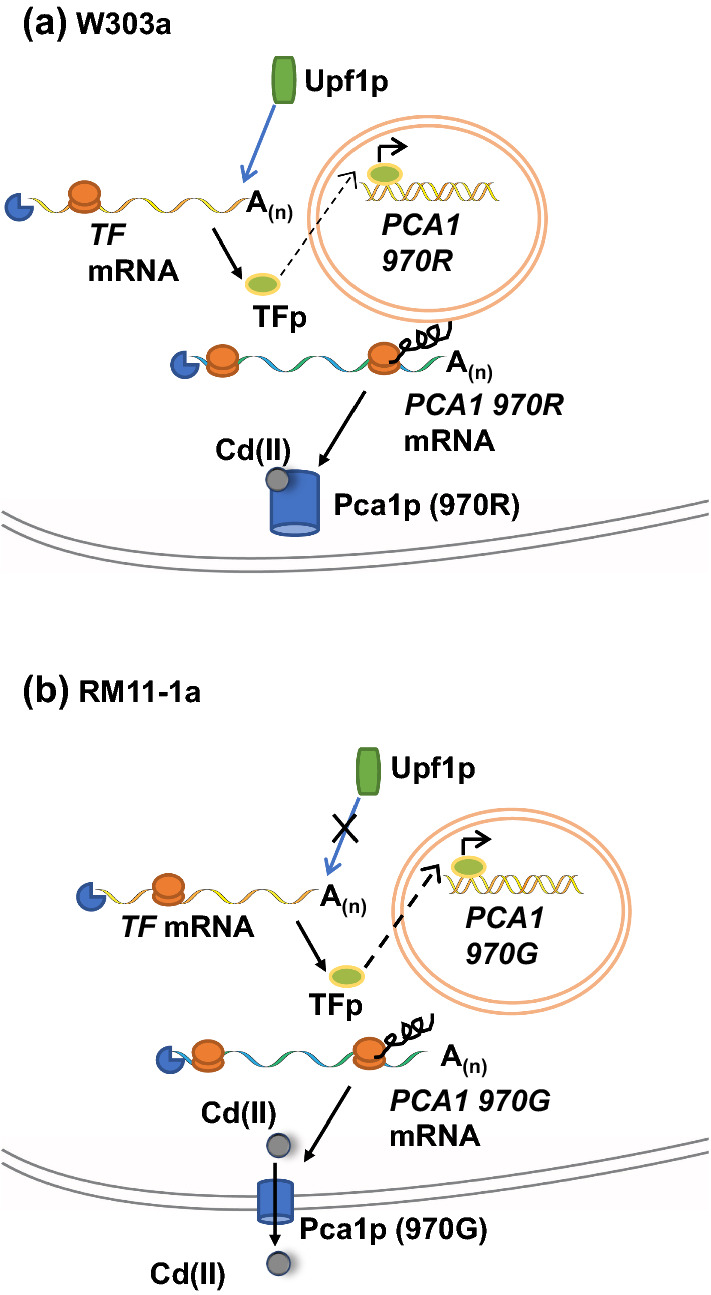
Schematic representation of different responses of *PCA1* alleles to environmental Cadmium in laboratory and natural yeast strains. **a** *PCA1 970R* mRNA from commonly used lab yeast strain W303a is regulated by NMD when grown in cadmium containing media. The Pca1p (970R) does not function to pump cadmium out from yeast cells. Pca1p accumulates in vesicle like components in this case. **b** *PCA1 970G* mRNA from RM11-1a is resistant to NMD mediated regulation in cadmium containing media. In media containing cadmium, *PCA1 970G* evades NMD-mediated regulation. In this case, more Pca1p is most likely translated and actively functions in pumping cadmium out. Consequently, yeast cells tolerate cadmium toxicity. TF, transcription factor

Notably, *PCA1* transcripts produced by the natural yeast strains RM11-1a and lab strain W303a were differentially regulated by NMD in the presence of Cadmium. *PCA1* 970R mRNAs encoded by W303a were regulated by NMD under all the conditions tested, including cadmium. On the other hand, *PCA1 970G* mRNAs from RM11-1a were regulated by NMD under all conditions tested except when the cells were grown in media containing cadmium, suggesting that *PCA1 970G* transcripts may evade NMD under inducing conditions (Fig. 2). In the presence of copper, the *PCA1 970R* mRNA from W303a was an indirect NMD target. (Wong et al. 2021b).

## The role NMD plays in iron homeostasis

As previously mentioned, Aft1p and Aft2p induce transcription of genes in the iron regulon during iron scarcity. These Aft1p and Aft2p target genes include proteins that have ferric and cupric reductase activity. Select mRNAs in these group are regulated by NMD in a manner specific to growth conditions (Peccarelli et al. 2019). *FRE1* and *FRE2* encode for the major ferric and cupric reductases in *S. cerevisiae,* and they transcribe mRNAs that are differentially regulated by NMD (Table 1). *FRE1* mRNA is induced under low copper and low iron levels, but this mRNA is not regulated by NMD under all the conditions examined. On the other hand, *FRE2* mRNAs are regulated by NMD under normal growth conditions and low copper levels, but not under low iron levels (Peccarelli et al. 2019). These results suggest that condition-specific NMD-mediated degradation of mRNAs can be selective and differential.

As previously stated, Toesca et al. 2011, reported that NMD degrades 5′-extended transcripts produced from cryptic upstream transcription from authentic promoters, and that the upstream transcription is responsible for the repression of metal homeostasis genes in conditions of metal repletion. Importantly, the CD-CUTS facilitate transcriptional repression of metal homeostasis genes such as *FIT3* and *ZRT1.* The *FIT3* and *ZRT1* CD-CUTs prevent the premature binding of the RNA polymerase, or of transcriptional activators such as Zap1p and Aft1p when these genes are transcriptionally repressed. The *FIT3* gene is involved in siderophore-iron transport facilitating.

NMD targets are distinguished from non-targets by an aberrant translation termination event that results in rapid degradation of NMD targets. It has been demonstrated that the essential Fe–S protein *Rli1* (*ABCE1* in humans), which is involved in ribosome biogenesis and recycling, is required for maintaining regulatory pathways including NMD (Zhu et al. 2020). In the absence of *Rli1*, unrecycled yeast ribosomes move into the 3′-UTR of the mRNA where they initiate translation aberrantly and indiscriminately displace trans factors bound to the mRNAs 3′-UTR. These displaced factors include proteins bound to adenosine and uridine-rich elements (AREs) in the 3′-UTR of mRNAs, such as Cth1p and Cth2p resulting in mis-regulation of iron homeostasis (Puig et al. 2005, 2008). Conversely, mis-regulation of iron homeostasis disrupts *Rli1* function, which in turn could disrupt NMD function because the translation termination event determines whether the NMD pathway will take place. Thus, NMD and iron homeostasis are also interlinked, and accurate regulation of both processes is essential for normal cellular function.

## Evolutionary aspect of NMD-mediated regulation of Bio-metal homeostasis and toxic metal detoxification

A study on the evolution of metal resistance in natural yeast populations found that cadmium tolerance was controlled primarily by the *PCA1* locus, which classifies cadmium resistance as the ancestral phenotype (Chang and Leu 2011). It is postulated that during yeast evolution, *PCA1* experienced several rounds of selective adaptation on the promoter sequence (Chang and Leu 2011). Notably, yeast cells overexpressing *PCA1* in a medium without cadmium suffered reduced fitness (Rad et al. 1994). As a result of a tradeoff between metal resistance and fitness, most *S. cerevisiae* populations contain an altered *PCA1* allele (Chang and Leu 2011). This study found that the enhancement of cadmium resistance can be largely attributed to mutations in the promoter sequence.

A further study proposed that the cadmium resistant strain was a gain-of-function mutation within *PCA1* Arg970gly, generating cadmium efflux and tolerance (Shiraishi et al. 2000). Furthermore, a phylogenetic analysis based on *PCA1* screened the two alleles of *PCA1 970G* and *PCA1 970R* among yeast populations and revealed that 970R was the ancestral allele while 970G evolved from that ancestral lineage (Wong et al. 2021). A single nucleotide difference at codon 970 is associated with response to cadmium (Wong et al. 2021). The 970G allele that facilitates tolerance to cadmium was the most common allele. These two studies demonstrate that the evolution of *PCA1* with regards to cadmium tolerance can be in different regions of the gene. Further, the studies suggest that the evolution of metal tolerance by different yeast strains can affect the regulation of mRNAs generated from different alleles by NMD.

## Conclusions

The importance of NMD in metal ion homeostasis and toxic metal detoxification is highlighted here. The NMD pathway regulates select mRNAs involved in magnesium, zinc, iron, and copper homeostasis, as well as cadmium detoxication. NMD is a translation dependent process that ensues once translation is terminated aberrantly, and metal ions can affect the reliability of translation. Bio-metal homeostasis is related to NMD mediated regulation of gene expression in the case of magnesium homeostasis (Johansson and Jacobson 2010), which facilitates NMD fidelity. Furthermore, translation and iron homeostasis are closely interconnected. Fe–S proteins such as *Rli1* (*ABCE1* in humans), are involved in ribosome biogenesis and recycling and are required for maintaining regulatory pathways including NMD. Further studies investigating the extent to which NMD regulates mRNAs involved in the adaptation of yeast cells to changes in levels of metal ions and the role these metals ions play in translation and its reliability will elucidate metal ion homeostasis and NMD. Additionally, there are likely other metal ion homeostatic mechanisms impacted by NMD that need to be explored.

In terms of specific mRNA targets regulated by NMD and involved in metal ion homeostasis, one of the questions that have arisen leads inquiries as to why there is variability in NMD-mediated regulation of the mRNAs that encode proteins with similar functions. For example, of the two copper binding metallothionein in yeast *CRS5* and *CUP1*, *CRS5* is regulated by NMD while *CUP1* is not. Moreover, mRNAs encoded by different alleles are differentially regulated by NMD based on environmental conditions. For example, *PCA1 970G* and *PCA1 970R* mRNAs are differentially regulated by NMD in the presence of cadmium. It would be significant to understand how prevalent this environmentally specific differential regulation of mRNAs by NMD is.
